# The Roles of Low-Density Lipoprotein Receptor-Related Proteins 5, 6, and 8 in Cancer: A Review

**DOI:** 10.1155/2019/4536302

**Published:** 2019-03-26

**Authors:** Zulaika Roslan, Mudiana Muhamad, Lakshmi Selvaratnam, Sharaniza Ab-Rahim

**Affiliations:** ^1^Institute of Medical and Molecular Biotechnology, Faculty of Medicine, Universiti Teknologi MARA, Cawangan Selangor, Kampus Sungai Buloh, 47000 Sungai Buloh, Selangor, Malaysia; ^2^Department of Biochemistry and Molecular Medicine, Faculty of Medicine, Universiti Teknologi MARA, Cawangan Selangor, Kampus Sungai Buloh, 47000 Sungai Buloh, Selangor, Malaysia; ^3^Jeffrey Cheah School of Medicine & Health Sciences, Monash University Malaysia, Jalan Lagoon Selatan, 47500 Bandar sunway, Selangor, Malaysia

## Abstract

Low-density lipoprotein receptor (LDLR) has been an object of research since the 1970s because of its role in various cell functions. The LDLR family members include LRP5, LRP6, and LRP8. Even though LRP5, 6, and 8 are in the same family, intriguingly, these three proteins have various roles in physiological events, as well as in regulating different mechanisms in various kinds of cancers. LRP5, LRP6, and LRP8 have been shown to play important roles in a broad panel of cancers. LRP5 is highly expressed in many tissues and is involved in the modulation of glucose-induced insulin secretion, bone development, and cholesterol metabolism, as well as cancer progression. Recently, LRP5 has also been shown to play a role in chondroblastic subtype of osteosarcoma (OS) and prostate cancer and also in noncancer case such as osteoporosis. LRP6, which has been previously discovered to share the same structures as LRP5, has also been associated with many cancer progressions such as human triple negative breast cancer (TNBC), primary chronic lymphocytic leukemia (CLL), nonsmall cell lung cancer (NSCL), lung squamous cell carcinoma (LSCC), and hepatocellular carcinoma (HCC). In addition to its role in cancer progression, LRP8 (apolipoprotein E receptor 2 [APOER2]) has also been demonstrated to regulate canonical Wnt/*β*-catenin signaling pathway whereby this pathway plays a role in cell migration and development. Therefore, this review aimed to elucidate the role of LRP 5, 6, and 8 in regulating the cancer progression.

## 1. Introduction

Michael Brown and Joseph Goldstein have discovered low-density lipoprotein receptor (LDLR) while they were searching for the molecular basis of familial hypercholesterolemia (FH) [1]. Through this discovery, a mutation in LDLR in FH patient's fibroblast cells was observed where it lacked high-affinity sites when compared to the normal cultured fibroblast cells. This work has landed both researchers their Nobel Prize in 1985. Approximately, one in 500 patients has this mutation and, to date, nearly 900 LDLR mutations were associated with FH. Following this finding, the LDLR gene encoding the receptor was cloned and sequenced. The LDLR facilitated the cellular uptake of the cholesterol-rich lipoprotein which formed the circulation by binding to two ligand molecules: apolipoprotein B (APOB) and apolipoprotein E (APOE), of LDL and *β*-very LDL particles in the normal cells [2].

## 2. The Family Members of Low-Density Lipoprotein-Related Receptor (LDLR)

The first vertebrate lipoprotein receptor discovered was human low-density lipoprotein receptor (LDLR) (Schneider et al., 1999). The family members include LDLR; LDLR-related protein 1 (LRP1), LRP2/glycoprotein330/megalin, very low-density lipoprotein (VLDL) receptor (VLDLR); LR11 (also known as sorLA); apolipoprotein E (apoE) receptor type 2 (apoER2, LRP8, LR7/8B); LRP3 (closely resembles ST7/LRP12), MEGF7/LRP4, LRP5, and LRP6, and LR32 (also called LRP1B) [3].

LDLR mammalian family members can be structurally subgrouped into at least four subgroups. The first group comprises LDLR, VLDLR, and LRP8. LRP1, LRP1B, and LRP2 belong to the second group. LRP5 and LRP6 are members of the third group, whereas DR11, LRP4, ST7, and LRP3 are in the fourth group. The early comparison of LDLR sequences revealed several conserved domains from different species. In addition, there are two examples of LDLR sequences: type A repeat (LDL-A) and type B repeat (LDL-B). LDL-A consists of approximately 40 amino acids residues, each harboring 6 paired cysteines in identical positions and a cluster of negatively charged residues. On the contrary, LDL-B contains four-amino-acid sequence of Tyr-Trp-Thr-Asp (YWTD) [4]. In addition, modular domain organisation was shown in family members of LDLR and characteristically contains one or multiple copies of a conserved cysteine-rich domain.

## 3. LDLR and Cancers

A number of studies have demonstrated the associations of LDLR in many cancers including liver cancer, leukemia, lung cancer, breast cancer, colorectal cancer, and prostate cancer [5, 6]. Naturally, cancer cells require higher uptake of cholesterol than normal cells whereby receptor-mediated endocytosis of serum LDL enhances the cholesterol content through LDLR [7]. Previous study has also suggested that lack of feedback of regulation of LDLR in prostate cancer (PC3 cells) provided an extra energy source to promote their uncontrolled growth [8]. The same study also reported that LDLR inhibits the cell proliferation of prostate cancer when induced with statins. It was also suggested that LDLR activates the signaling pathways involved in cell growth, inflammation, and cellular transformation. LDLR was also reported to have protumorigenic effect [9] and promote proliferation and cancer progression by the migration of the tumor cells [10, 11].

In addition, mutations of this protein were shown to cause defects in synthesis, transport, binding, internalisation, and recycling of LDLR proteins [12]. In another study, the LDLR expression in human breast cancers was inversely linked with their survival [13]. The study also reported that LDLR is expressed in human triple negative cell line and human breast cancer cell lines. In addition, MDA-MB-231 cells were reported to have high LDLR expression than the estrogen receptor positive MCF7 or the nontumorigenic MCF-10A cell lines.

Previous study also showed the association of LDLR in Glioblastoma (GBM). GBM is the most common malignant primary brain tumor and one of the most lethal cancers. The protein expression of LDLR has enhanced the uptake of extracellular cholesterol in GBM clinical samples, xenograft models, and cell lines [14]. This study revealed that Liver X receptor (LXR) potently induces tumor cell death* in vivo*, an effect highly correlated with decreased LDLR protein expression and increased ABCA1-dependent cholesterol efflux.

Besides, LDL and LDLR were also identified as prognostic factors in pancreatic adenocarcinoma. Their expressions were reported to be negatively correlated with clinical outcome as well as in small cell lung cancer (SCLC) [7]. To date, LDLR family members have been reported in various cancers; however, their roles were not fully understood as each family member has different roles and functions. Therefore, this review will focus on LRP5, 6, and 8 in terms of their relation and mechanism towards cancer progression.

## 4. LRP5 Structure and Mechanism in Cancer Progression

LRP5 was highly expressed in many tissues [15]. It was discovered unexpectedly during the attempts to identify the nature of IDDM locus on the chromosome 11q13. Since then, it has been an important concept in the study of the insulin dependent diabetes mellitus (IDDM) whereby LRP5 expression was suggested to be associated with susceptibility to diabetes. In addition, many studies have reported that LRP5 gene is involved in the cholesterol metabolism and bone development.

**Figure 1:**
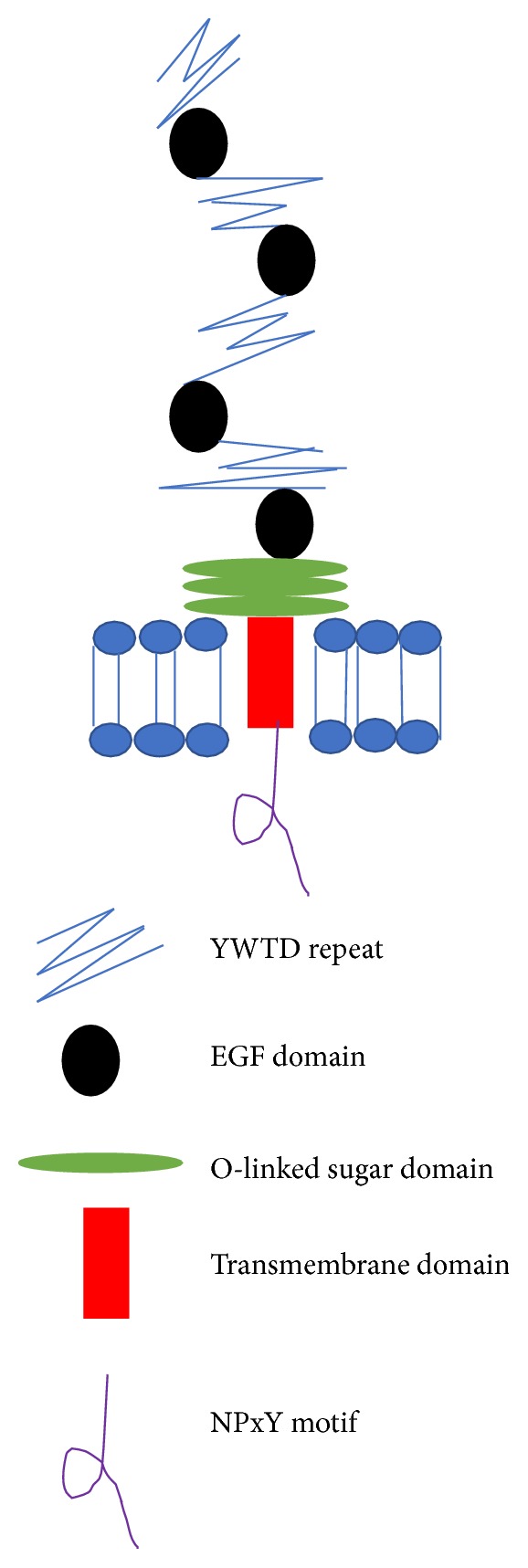
The diagram showed LRP5/6 that consists of YWTD repeats and three O-linked sugar domain (LA) repeats. It was recognised as cell surface endocytic receptors in the LDL-receptor family which bind and internalise extracellular ligands for degradation by lysosomes (adapted from Bovenshen, 2010).

LRP5 has the same features as the type-1 membrane protein which consists of 1600 residues long. LRP5 has extracellular domains which are organised exactly as a portion of LRP (Figure 1). It consists of YWTD repeats and 3 LA repeats [16]. It was recognised as cell surface endocytic receptors in the LDL-receptor family which bind and internalise extracellular ligands for degradation by lysosomes.

LRP5 was identified as Wnt canonical signaling pathway single-pass transmembrane coreceptors by many researchers. The Wnt proteins secreted modulate cell proliferation and cell fate during the oncogenesis and embryonic development through activation of receptor-mediated signaling pathways.

**Figure 2:**
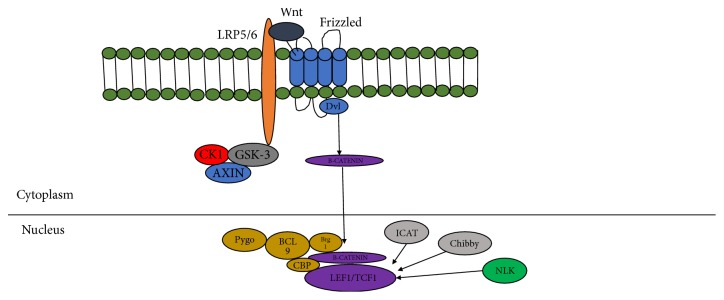
The binding of Wnts to FZD and LRP5 leads to the downregulation of GSK-3 activity and initiates the canonical Wnt/beta-catenin signaling cascade (adapted from Cell Signaling Technology).

The binding of Wnt ligands to the cell surface receptors frizzled (FZD) and LRP5 leads to cytoplasmic complex blockade which consists of glycogen-synthase-kinase-3-*β* (GSK3*β*), adenomatous polyposis coli (APC), and axin 2 (AXIN2) [17]. The binding of Wnts to FZD and LRP5 leads to the downregulation of GSK-3 activity and initiates the canonical Wnt/beta-catenin signaling cascade (Figure 2). There are also various other proteins that interact and/or regulate LRP5. They are R-spondin, Kremen, Complement C1q, Apolipoprotein E, Biglycan, LRP4, and Phosphoregulation LRP5/6 [18].

The mutation of LRP5 has been said to be associated with osteoporosis and the change in bone mass syndrome. Furthermore, previous study has shown that the conditional deletion of the LRP5 gene in mice resulted in an enhanced bone formation [19]. Another study also suggested that LRP5 expression was stimulated by bone morphogenic protein 2 (BMP2) and downregulates beta-transducin repeats-containing protein (*β*-Trcp). This has led to the *β*-catenin stabilisation and promotes osteogenic differentiation in normal cells [20].

In relation to cancer progression, LRP5 has been shown to be involved in mediating Wnt/*β*-catenin signaling in skeletal metastasis prostate cancer (PC) due to the increased level of Wnt-1 and *β*-catenin proteins in both PC cell lines and primary specimens [19]. The presence of LRP5 has also been linked significantly with tumor metastasis such as chondroblastic subtype of OS [21]. In this study, patients with positive LRP5 expression showed a trend on decreased of event-free survival. However, there is no significant association found between the LRP5 expression with age, gender, site of disease, site of metastasis, or degree of chemotherapy-induced tumor necrosis. In addition, the sequencing of exon 3 of LRP5 in 10 OS patient-derived cell cultures showed no activating mutation of LRP5 [21]. In a separate study, the decreased cell invasion and motility were observed in OS cell lines where dominant-negative LRP5 and Dickkopf-3 (Dkk-3) were found to be mutated [22]. These results showed that LRP5 expression is a common event in OS and strongly suggest a role for LRP and Wnt signaling in the pathobiology and progression of this disease [21].

Interestingly, in contradiction to the above studies, recent study has shown that dominant-negative LRP5 failed to block OS formation, metastatic disease, and even maintenance of Wnt signaling in mice [17]. This contradicting data suggest that the cancer progression through Wnt signaling pathway by LRP5 might not be as direct as we think but could be depending on the degree of the cancer progression or the heterogeneity of the cancer type itself.

## 5. LRP6 Structure and Mechanism in Cancer Progression

The low-density lipoprotein receptor-related protein 6 (LRP6) is one of the low-density lipoprotein receptor (LDLR) family members that shared almost the same structures with LRP5 (Figure 1). LRP6 displays a high level of grouping homology where it shared 73% and 64% sequence identity with LRP5 in their extracellular and intercellular domains, respectively. LRP6 is a single-pass transmembrane receptor with extracellular domain (containing four tandem *β*-propeller/epidermal growth factor repeats) and followed by three LDLR type A repeats [23]. With this, the link of these two receptors was anticipated. However, these two receptors were reported to be unique in their functions* in vivo *[24].

LRP6 was first discovered while screening the mouse liver cDNA library with an EST W20530 sequence [16]. It was initially reported to be more active than LRP5 in stimulating signaling activity. Human LRP6 gene was located in chromosome 12p13.2 with 150kb in length and 23 exons is highly conserved in all species. This gene encodes for a protein of 1613 amino acids which is a signal transmembrane protein. LRP6 receptor, like LRP5, also interacts with frizzled (FZD) family which has seven transmembrane receptors to activate the Wnt/*β*-catenin signaling pathway (Figure 2). Because LRP6 roles are similar to LRP5 where it acts as coreceptor, it transduces a signal across the plasma membrane that resulted in the activation of the Dishevelled (DVL).

LRP6 has been reported in a broad panel of cancers including breast cancer, prostate cancer, hepatocellular carcinoma, and retinoblastoma. The expression of LRP6 was found to be upregulated in these cancers [25, 26] and altered LRP6 leads to abnormal Wnt protein activation. In one study, the level of LRP6 decreased in Wnt signaling pathway when Salinomycin was given to the primary chronic lymphocytic leukemia (CLL) [27]. Salinomycin is used to kill breast cancer stem and it was also reported to be the inhibitor of Wnt/*β*-catenin signaling by inducing LRP6 degradation [28]. Besides Salinomycin, Niclosamide was also reported to suppress LRP6 expression in TNBC MDA-MB-231 cells and ER-positive breast cancer T-47D cells where it inhibited breast cancer cell proliferation [29].

In addition, recent study has demonstrated the upregulation of LRP6 expression in both human triple negative breast cancer (TNBC) cell lines and patients. Besides, the knockout of LRP6 expression has fundamentally diminished the cell migration and invasion of TNBC MDA-MB-231 and BT549 [26]. In another study, overexpressed LRP6 was also observed in a subpopulation of the human breast cancers suggesting that the Wnt signaling pathway activation by the overexpressed LRP6 was enough to induce the formation of breast cancer [30].

LRP6 polymorphisms were validated to play a role in the predisposition of the nonsmall cell lung cancer (NSCLS) in previous study. These polymorphisms were also found associated with a reduced risk of lung squamous cell carcinoma (SCC) where LRP6 rs6488507 synergistically increased the risk of NSCLS in tobacco smokers in Chinese populations [31]. Likewise, LRP6 was found in hepatocellular carcinoma (HCC) whereby oxaliplatin-pretreated hepatocellular carcinoma showed the improvement of its stemness and increased expression of LRP6 and connective tissue development factor (CCN2) [32]. In this study, LRP6 was suggested to be positively related to malignant phenotype and poor prognosis in human HCC, where the LRP6 could contribute to the upregulation of CCN2. This resulted in enhanced stemness phenotype of HCC.

From these previous studies, it is evidently showed that mutations in LRP6 have been linked to a wide variety of cancers. It also showed its critical role in Wnt signaling pathway which confirmed that the LRP6 variants are related to the risk of cancer and tumor progression.

## 6. LRP8 Structure and Mechanism in Cancer Progression

**Figure 3:**
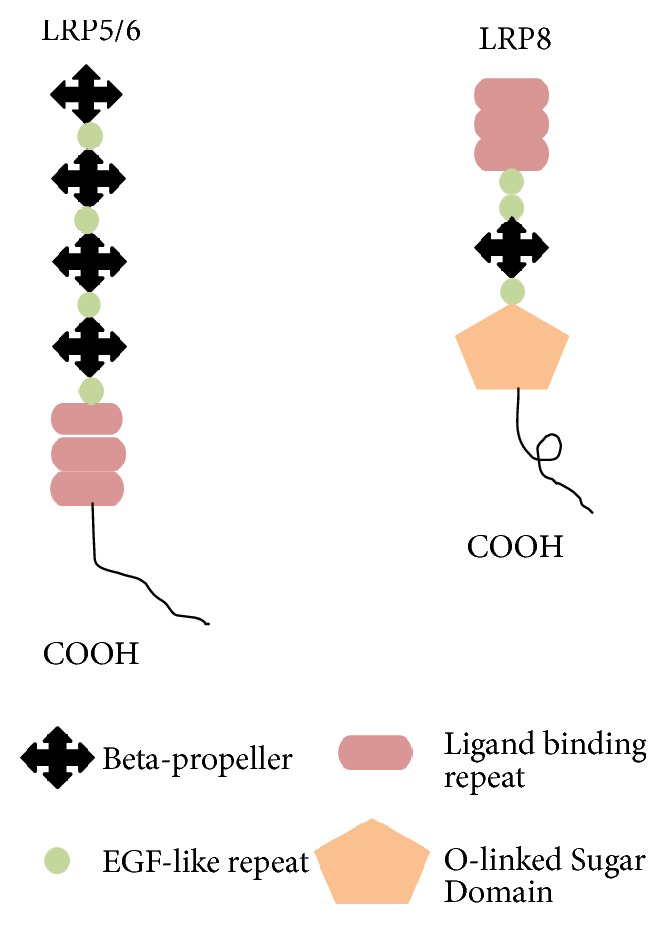
This figure showed schematic structure of LRP5, LRP6, and LRP8. There are repetition and almost similar groups presented in all structures such as beta-propeller, EGF-like repeat, and ligand binding repeat.

Apolipoprotein E receptor 2 (APOER2) also known as low-density lipoprotein receptor-related protein 8 (LRP8) is also classified under the LDLR family. LRP8 consists of seven conserved LDL-A repeats followed by three EGF receptor-like domains and *β*-propeller motif. Unlike LRP5/6 which have four YWTD *β*-propeller, LRP8 has only one *β*-propeller motif (Figure 3). LRP8 also has one NPxY motif in the intracellular domain which intervenes in the association with the phosphotyrosine-authoritative (PTB) domain-containing proteins [33]. LRP8 was first identified in the brain and has also been found abundantly in placenta, ovaries, and epididymis.

LRP8 and the VLDL receptor are high affinity receptors for Reelin, a large extracellular matrix protein. Reelin is a vast, secreted extracellular matrix glycoprotein that promotes DAB1-mediated recruitment of PI3K and GSK3B to LRP8 ICD [34]. It was shown to regulate neuronal differentiation and migration by enhancing Reelin endocytosis.

Previous study has reported that LRP8 might have been associated with Alzheimer's disease when LRP8 gene polymorphism occurred. Besides, the double-knockout mice of LRP8 and VLDLR displayed disorganised neurons and cortical overlay which influenced the brain development [33].

LRP8 has also been reported as a positive regulator of the Wnt/*β*‐catenin signaling pathway. It interacted with endogenous Axin when cells were challenged with Wnt3. It was also reported as a novel membrane-associated regulator of canonical Wnt/*β*-catenin signaling and promotes Wnt3a-induced osteoblast differentiation [33].

In addition, LRP8 is also one of the four ApoE receptors identified in melanoma cells with VLDLR, LRP1, and LDLR being the other three receptors [35]. Both LRP8 and VLDLR additionally demonstrated the most predictable inhibitory impact in siRNA screen and were therefore selected for further mechanistic studies* in vivo* and* in vitro*. This study showed that LRP8 and VLDLR have the critical survival factors for TNBC specifically when under starvation and enable cells to maintain metabolic activity [36].

In another study, LRP8 was knocked down to investigate the molecular mechanisms in ApoE suppressed metastasis. In melanoma cancer, endothelial LRP8 was knocked down selectively and this resulted in significantly abrogating the suppressed endothelial recruitment phenotype induced by miRNA silencing. Here, LRP8 acts as the endothelial mediator of miRNA/ApoE-dependent effects on endothelial recruitment. Besides, LRP8 was also discovered to be endogenous suppressor of prometastasis phenotype in melanoma cancer [35].

## 7. Conclusion

It is clear that LRP5 and 6 have a functional role in cancer cell progression as coreceptors of the Wnt/*β*-catenin pathway. In addition LRP8 has also gained much attention in its role as one of the coreceptors involved in regulating cancer progression. However, most of the published data were at the preliminary stage. Therefore, further investigations are in need to validate the currently reported biomarkers in LRP6, 7, and 8 especially in unmasking the underlying mechanism of these proteins in regulating cancer progression through Wnt/*β*-catenin pathway.
